# Effects of Comprehensive Stroke Care Capabilities on In-Hospital Mortality of Patients with Ischemic and Hemorrhagic Stroke: J-ASPECT Study

**DOI:** 10.1371/journal.pone.0096819

**Published:** 2014-05-14

**Authors:** Koji Iihara, Kunihiro Nishimura, Akiko Kada, Jyoji Nakagawara, Kuniaki Ogasawara, Junichi Ono, Yoshiaki Shiokawa, Toru Aruga, Shigeru Miyachi, Izumi Nagata, Kazunori Toyoda, Shinya Matsuda, Yoshihiro Miyamoto, Akifumi Suzuki, Koichi B. Ishikawa, Hiroharu Kataoka, Fumiaki Nakamura, Satoru Kamitani

**Affiliations:** 1 Department of Neurosurgery, Graduate School of Medical Sciences, Kyushu University, Fukuoka, Japan; 2 Department of Preventive Medicine and Epidemiologic Informatics, National Cerebral and Cardiovascular Center, Suita, Japan; 3 Clinical Research Center, National Hospital Organization, Nagoya Medical Center, Nagoya, Japan; 4 Integrative Stroke Imaging Center, National Cerebral and Cardiovascular Center, Suita, Japan; 5 Department of Neurosurgery, Iwate Medical University, Morioka, Japan; 6 Chiba Cardiovascular Center, Ichihara, Japan; 7 Department of Neurosurgery, Kyorin University, Mitaka, Japan; 8 Showa University Hospital, Tokyo, Japan; 9 Department of Neurosurgery, Nagoya University, Nagoya, Japan; 10 Department of Neurosurgery, Nagasaki University, Nagasaki, Japan; 11 Department of Cerebrovascular Medicine, National Cerebral and Cardiovascular Center, Suita, Japan; 12 Department of Preventive Medicine and Community Health, University of Occupational and Environmental Health, Kita-Kyushu, Japan; 13 Research Institute for Brain and Blood Vessels, Akita, Japan; 14 Center for Cancer Control and Information Services, National Cancer Center, Tokyo, Japan; 15 Department of Neurosurgery, National Cerebral and Cardiovascular Center, Suita, Japan; 16 Department of Public Health, The University of Tokyo, Tokyo, Japan; Weill Cornell Medical College, United States of America

## Abstract

**Background:**

The effectiveness of comprehensive stroke center (CSC) capabilities on stroke mortality remains uncertain. We performed a nationwide study to examine whether CSC capabilities influenced in-hospital mortality of patients with ischemic and hemorrhagic stroke.

**Methods and Results:**

Of the 1,369 certified training institutions in Japan, 749 hospitals responded to a questionnaire survey regarding CSC capabilities that queried the availability of personnel, diagnostic techniques, specific expertise, infrastructure, and educational components recommended for CSCs. Among the institutions that responded, data on patients hospitalized for stroke between April 1, 2010 and March 31, 2011 were obtained from the Japanese Diagnosis Procedure Combination database. In-hospital mortality was analyzed using hierarchical logistic regression analysis adjusted for age, sex, level of consciousness on admission, comorbidities, and the number of fulfilled CSC items in each component and in total. Data from 265 institutions and 53,170 emergency-hospitalized patients were analyzed. Mortality rates were 7.8% for patients with ischemic stroke, 16.8% for patients with intracerebral hemorrhage (ICH), and 28.1% for patients with subarachnoid hemorrhage (SAH). Mortality adjusted for age, sex, and level of consciousness was significantly correlated with personnel, infrastructural, educational, and total CSC scores in patients with ischemic stroke. Mortality was significantly correlated with diagnostic, educational, and total CSC scores in patients with ICH and with specific expertise, infrastructural, educational, and total CSC scores in patients with SAH.

**Conclusions:**

CSC capabilities were associated with reduced in-hospital mortality rates, and relevant aspects of care were found to be dependent on stroke type.

## Introduction

In Japan, stroke is the third-leading cause of death, as well as a leading cause of long-term disability. Almost 270,000 individuals in Japan have a new or recurrent stroke each year, and nearly 120,000 individuals die following a stroke [1]. In 2000, the Brain Attack Coalition discussed the concept of stroke centers and proposed two types of centers: comprehensive [2] and primary [3]. Most patients with stroke can be appropriately treated at a primary stroke center (PSC), and the Joint Commission has established programs for certifying PSCs and measuring their performance [4]. The concept and recommended key components of comprehensive stroke centers (CSCs) enable intensive care and specialized techniques that are not available at most PSCs [2], [5]. Although stroke performance measures have been developed to monitor and improve quality of care, a substantial proportion of patients do not receive effective treatments, and population-based studies have been called for to evaluate the successful translation of evidence-based medicine into clinical practice [4] Organized stroke unit care is a form of in-hospital care provided by nurses, doctors, and therapists who work as a coordinated team specialized in caring for patients with stroke [6]. The effectiveness of organized stroke care has been reported for different ischemic stroke subtypes in the organized care index (OCI) using data from the Registry of the Canadian Stroke Network [7], [8]. However, the effectiveness of CSC capabilities on the mortality of patients with ischemic and hemorrhagic stroke remains uncertain. In this study, we examined whether CSC capabilities influence in-hospital mortality for all types of stroke in a real-world setting by using data from the J-ASPECT nationwide stroke registry (obtained from the Japanese Diagnosis Procedure Combination [DPC]-based Payment System) [9].

## Methods

Of the 1,369 certified training institutions of the Japan Neurosurgical Society, the Japanese Society of Neurology, and/or the Japan Stroke Society, 749 hospitals responded to a questionnaire survey regarding CSC capabilities. The CSC capabilities were assessed using 25 items specifically recommended for CSCs [2] that were divided into 5 components regarding (1) personnel (seven items: board-certified neurologists, board-certified neurosurgeons, board-certified endovascular physicians, board-certified physicians in critical care medicine, board-certified physicians in physical medicine and rehabilitation, personnel in rehabilitation therapy, and stroke rehabilitation nurses), (2) diagnostic techniques (six items: 24 hours/day, 7 days/week [24/7] availability of computed tomography [CT], magnetic resonance imaging [MRI] with diffusion-weighted imaging, digital cerebral angiography, CT angiography, carotid duplex ultrasound, and transcranial Doppler), (3) specific expertise (five items: carotid endarterectomy, clipping of intracranial aneurysms [IAs], removal of intracerebral hemorrhage [ICH], coiling of IAs, and intra-arterial reperfusion therapy), (4) infrastructure (five items: stroke unit, intensive care unit, operating room staffed 24/7, interventional services coverage 24/7, and stroke registry), and (5) educational components (two items: community education and professional education). A score of 1 point was assigned if the hospital met each recommended item, yielding a total CSC score of up to 25. The scores were also summed for each component (subcategory CSC score). The impact of specific aspects of acute stroke care (monitoring, early rehabilitation, admission to stroke care unit [SCU], acute stroke team, the organized stroke care index [7], existence of a tissue plasminogen activator [t-PA] protocol, number of t-PA cases/year, and number of acute stroke cases/year) on stroke mortality was also examined. This survey was completed by neurosurgeons or neurologists in the responding hospitals and returned by mail. Any incomplete answers were completed in follow-up phone interviews with the neurosurgeons or neurologists of the study group. The English version of the survey is shown in File S1.

This cross-sectional survey used the DPC discharge database from participating institutions in the J-ASPECT Study. The DPC is a mixed case patient classification system that was launched in 2002 by the Ministry of Health, Labor, and Welfare of Japan and was linked with a lump-sum payment system [9]. Of the 749 hospitals that responded to the institutional survey regarding CSC capabilities, 265 agreed to participate in the DPC discharge database study (File S2). Computer software was developed to identify patients hospitalized because of acute stroke from the annual de-identified discharge database by using the International Classification of Diseases (ICD)-10 diagnosis codes related to ischemic stroke (I63.0-9), nontraumatic ICH (ICH: I61.0-9, I62.0-1, and I62.9), and subarachnoid hemorrhage (SAH: I60.0-9). Because of major differences in their typical prognosis, patients with transient ischemic attack were excluded. Patients hospitalized because of ischemic and hemorrhagic stroke between April 1, 2010 and March 31, 2011 were included; however, patients with scheduled admissions were excluded from analysis. The following data were collected from the database: unique identifiers of hospitals, patients' age and sex, diagnoses, comorbidities at admission, in-hospital use of medications (antihypertensive agents, oral hypoglycemic agents, insulin, antihyperlipidemic agents, statins, anticoagulant agents, or antiplatelet agents), smoking, arrival by ambulance or not, level of consciousness at admission according to the Japan Coma Scale [10], and discharge status. The Japan Coma Scale [11] was originally published in 1974, the same year as the Glasgow Coma Scale (GCS) [12], and it remains one of the most popular grading scales for assessing impaired consciousness among health care professionals and personnel for emergency medical services in Japan. Grading with the 1-, 2-, and 3-digit codes corresponds to the following statuses: 1) the patient is awake in the absence of any stimulation, 2) the patient can be aroused but reverts to the previous state after the cessation of stimulation, and 3) the patient cannot be aroused even by forceful mechanical stimulation. Each specific digit status is further subdivided into three levels: 1-digit code into 1, 2, and 3; 2-digit code into 10, 20, and 30; and 3-digit code into 100, 200, and 300 (Table S1). In addition to these nine grades, a normal level of consciousness is graded as zero. Consciousness level on admission was determined by the physician and data on all medication use was collected electronically from the claim data. Comorbidity was determined primarily from the ICD-10 code, but was also checked against what medications and procedures the patient was receiving/undergoing, to see if these were compatible with the code data. Smoking was defined by the physician's record, which rated patients as active or inactive smokers. In-hospital mortality, defined as death by the time of discharge from the hospital, was analyzed with the total and subcategory CSC scores using hierarchical logistic regression analysis adjusted for age, sex, Japan Coma Scale score, comorbidities, and institutional difference.

### Ethics Statement

This research plan was designed by the authors and approved by the Institutional Review Board of the National Cerebral and Cardiovascular Center, which waived the requirement for individual informed consent.

### Statistical Analysis

We used hierarchical logistic regression models [13], [14] to estimate odds ratios (ORs) for in-hospital mortality. Each model had two levels of hierarchy (hospital and patient) while considering the random effects of hospital variation, as well as fixed effects of CSC score and patient effects of age, sex, and level of consciousness. The total score and each subcategory score were analyzed separately. We also divided CSC score into quintiles and analyzed the trend with the Cochran-Armitage trend test. The difference between participating and non-participating hospitals in the DPC discharge study was determined by Wilcoxon rank-sum test. The analyses were performed using SAS version 9.2 (SAS Institute Inc., Cary, NC, USA) and STATA version 12 (STATA Corp, College Station, TX, USA).

## Results

A total of 265 hospitals participated in this study. The number and percentage of the participating hospitals with the recommended items of CSC capabilities are shown in Table 1. The distribution of total CSC scores ranged from 1 to 23 (mean: 15.4, median: 14 standard deviation [SD]: 4.2, interquartile range [IQR]: 11–18). Because we initially sent the CSC questionnaire to 749 hospitals, we sought to determine whether there was a selection bias in stroke care capabilities that could have impacted which hospitals returned the questionnaire. We found that such a bias did exist; in fact, the total CSC scores, subcategory CSC scores and annual volume of t-PA infusion, with the exception of the diagnostic techniques and education/research subcategories, were significantly higher for the participating hospitals than for the non-participating hospitals (Table 2).

**Table 1:** Number and percentage of participating hospitals (n = 265) with the recommended items of comprehensive stroke care capabilities.

Components	Items	*n*	%
Personnel	Neurologists	143	54.0
	Neurosurgeons	251	94.7
	Endovascular physicians	118	44.5
	Critical care medicine	65	24.5
	Physical medicine and rehabilitation	42	15.8
	Rehabilitation therapy	265	100
	Stroke rehabilitation nurses	38	14.6
Diagnostic techniques	CT	264	99.6
	MRI with diffusion	237	89.4
	Digital cerebral angiography	226	85.6
	CT angiography	234	88.3
	Carotid duplex ultrasound	102	38.5
	TCD	53	20.2
Specific expertise	Carotid endarterectomy	231	87.2
	Clipping of intracranial aneurysm	250	94.3
	Hematoma removal/draining	253	95.5
	Coiling of intracranial aneurysm	153	57.7
	Intra-arterial reperfusion therapy	199	75.1
Infrastructure	Stroke unit	55	20.8
	Intensive care unit	169	63.8
	Operating room staffed 24/7	185	70.0
	Interventional services coverage 24/7	122	46.0
	Stroke registry	109	41.8
Education	Community education	147	55.7
	Professional education	171	64.8

CT, computed tomography; MRI, magnetic resonance imaging; TCD, transcranial Doppler.

**Table 2:** Demographics of the study cohort according to the Diagnosis Procedure Combination (DPC) discharge database study in a comparison of hospitals that agreed to participate in the present study and those that did not.

	Participating hp	Non-participating hp	P value#
	(n = 265)	(n = 484)	
Hospital characteristics (CSC scores)			
Total score (25 items)	15.4±4.2	13.5±4.6	<0.001
Personnel (7 items)	3.5±1.2	3.1±1.3	<0.001
Diagnostic techniques (6 items)	4.2±1.2	3.9±1.3	0.002
Specific expertise (5 items)	4.0±1.4	3.6±1.6	<0.001
Infrastructure (5 items)	2.4±1.4	1.9±1.4	<0.001
Education (2 items)	1.2±0.8	1.0±0.8	0.002
Number of beds, n (%)			
20–49	3 (1.1)	13 (2.7)	<0.001
50–99	9 (3.4)	21 (4.3)	
100–299	66 (24.9)	166 (34.3)	
300–499	97 (36.6)	163 (33.7)	
500–	90 (34.0)	117 (24.2)	
Annual stroke cases, n (%)			
0–49	8 (3.0)	43 (8.9)	0.003
50–99	31 (11.7)	47 (9.7)	
100–199	56 (21.1)	143 (29.5)	
200–299	67 (25.3)	88 (18.2)	
300–	92 (34.7)	136 (28.1)	
Annual volume of t-PA infusion	8.3	6.4	0.002

# Wilcoxon rank-sum test.

CSC, comprehensive stroke center.

Hp, hospital.

Data from 265 institutions and 53,170 emergency-hospitalized patients (age in years, mean ±SD: 72.5±13.1; male: 55.2%) were analyzed. Patient demographics according to stroke type at the time of diagnosis are shown in Table 3. The study cohort included 32,671 patients with ischemic stroke (age: 74.4±12.2 years; male: 57.6%), 15,699 with ICH (age: 70.7±13.5 years; male: 57.5%), and 4,934 with SAH (age: 64.7±14.8 years; male: 32.1%). Use of antihypertensive agents, antidiabetic agents, antihyperlipidemic agents, and antiplatelet agents is also shown in Table 3. Almost 60% of the patients arrived by ambulance, with the incidence ranging from 77.6% for SAH to 53.1% for ischemic stroke. These rates of arrival by ambulance based on stroke type were in accordance with different degrees of stroke severity, as reflected by level of consciousness. Hospital characteristics shown by total and subcategory CSC scores did not reveal any significant differences with respect to stroke type.

**Table 3:** Demographics of the patient study cohort at the time of diagnosis and hospital characteristics according to stroke type.

	Total	Ischemic Stroke	Intracerebral hemorrhage	Subarachnoid hemorrhage
	(n = 53,170)	(n = 32,671)	(n = 15,699)	(n = 4,934)
Male, n (%)	29,353 (55.2)	18,816 (57.6)	9,030 (57.5)	1,584 (32.1)
Age, mean years ±SD	72.5±13.1	74.4±12.2	70.7±13.5	64.7±14.8
Hypertension, n (%)	39,918 (75.1)	22,531 (69.0)	13,281 (84.6)	4,229 (85.7)
Diabetes Mellitus, n (%)	13,725 (25.8)	9,318 (28.5)	3,278 (20.9)	1,174 (23.8)
Hyperlipidemia, n (%)	15,015 (28.2)	11,104 (34.0)	2,529 (16.1)	1,412 (28.6)
Smoking (n = 4,4842)	12,761 (24.0)	8,188 (25.1)	3,540 (22.5)	1,074 (21.8)
Medications during hospitalization				
Antihypertensive agent	34,136 (64.2)	17,694 (54.2)	12,537 (79.9)	4,019 (81.5)
Anti-renin-angiotensin system agent	19,881 (37.4)	10,262 (31.4)	8,280 (52.7)	1,410 (28.6)
Ca channel antagonist	25,984 (48.9)	10,469 (32.0)	11,719 (74.6)	3,903 (79.1)
Sympathetic antagonist	6,334 (11.9)	3,821 (11.7)	2,172 (13.8)	364 (7.4)
*β-blocker, α,β-blocker	4,357 (8.2)	3,048 (9.3)	1,133 (7.2)	188 (3.8)
α-blocker	2,374 (4.5)	953 (2.9)	1,232 (7.8)	200 (4.1)
Diuretic agent	9,950 (18.7)	5,860 (17.9)	3,074 (19.6)	1,049 (21.3)
Loop diuretic	7,434 (14.0)	4,609 (14.1)	1,912 (12.2)	940 (19.1)
Other diuretic	4,425 (8.3)	2,527 (7.7)	1,653 (10.5)	255 (5.2)
Antidiabetic agent	10,295 (19.4)	6,784 (20.8)	2,473 (15.8)	1,075 (21.8)
Insulin	7,654 (14.4)	4,597 (14.1)	2,044 (13.0)	1,046 (21.2)
Oral antidiabetic agent	5,749 (10.8)	4,459 (13.6)	1,110 (7.1)	197 (4.0)
Antihyperlipidemic agent	12,387 (23.3)	9,264 (28.4)	1,839 (11.7)	1,310 (26.6)
Statin	10,099 (19.0)	7,840 (24.0)	1,366 (8.7)	912 (18.5)
Antiplatelet agent	23,635 (44.5)	21,746 (66.6)	625 (4.0)	1,298 (26.3)
Aspirin	11,929 (22.4)	11,119 (34.0)	378 (2.4)	447 (9.1)
Japan Coma Scale				
0, n (%)	19,635 (36.9)	15,027 (46.0)	3,620 (23.1)	1,024 (20.8)
1-digit code, n (%)	19,371 (36.4)	12,375 (37.9)	5,934 (37.8)	1,117 (22.6)
2-digit code, n (%)	6,937 (13.0)	3,396 (10.4)	2,705 (17.2)	852 (17.3)
3-digit code, n (%)	7,227 (13.6)	1,873 (5.7)	3,440 (21.9)	1,941 (39.3)
Emergency admission by ambulance, n (%)	31,995 (60.2)	17,336 (53.1)	10,909 (69.5)	3,830 (77.6)
Average days in hospital (range)	21 (11–40)	20 (12–38)	22 (10–43)	30 (12–54)
Hospital characteristics (CSC scores)				
Total score (25 items)		16.7±3.8	16.8±3.4	17.1±3.4
Personnel (7 items)		3.7±1.2	3.7±1.2	3.8±1.2
Diagnostic techniques (6 items)		4.4±1.1	4.5±1.0	4.5±1.0
Specific expertise (5 items)		4.4±1.0	4.4±0.9	4.5±0.8
Infrastructure (5 items)		2.8±1.3	2.9±1.3	2.9±1.3
Education (2 items)		1.4±0.8	1.4±0.8	1.4±0.8

CSC, comprehensive stroke center.

*A composite variable with a pure beta antagonist and a mixed alpha/beta adrenergic antagonist (e.g., labetalol).

Overall, mortality rates were 7.8% for ischemic stroke, 16.8% for ICH, and 28.1% for SAH. Table 4–6 show the results of a hierarchical logistic regression analysis of these data. Mortality of patients with ischemic stroke was significantly correlated with male sex (OR = 1.23), age (10 incremental years, OR = 1.4), and level of consciousness (1-digit code: OR = 2.4, 2-digit code: OR = 7.46, 3-digit code: OR = 21.62, versus zero [normal consciousness {control}]) as patient characteristics, and total CSC score (OR = 0.97) adjusted for age, sex, and level of consciousness as a hospital characteristic (Table 4). Mortality of patients with ICH was also significantly correlated with male sex (OR = 1.72), age (10 incremental years, OR = 1.36), and level of consciousness (1-digit code: OR = 1.45, 2-digit code: OR = 4.22, 3-digit code: OR = 49.59, versus zero as control) as patient characteristics and total CSC score (OR = 0.97) adjusted for age, sex, and level of consciousness as a hospital characteristic (Table 5). Mortality of patients with SAH was likewise significantly correlated with male sex (OR = 1.39), age (10 incremental years, OR = 1.37), and level of consciousness (2-digit code: OR = 2.01, 3-digit code: OR = 17.12, versus zero as control) as patient characteristics and total CSC score (OR = 0.95) adjusted for age, sex, and level of consciousness as a hospital characteristic. Therefore, total CSC score was independently associated with in-hospital mortality for all stroke types after adjusting for age, sex, and stroke severity (Table 6). The impact of total CSC score on in-hospital mortality for ischemic stroke and ICH remained significant after adjustment for age, sex, severity of stroke, and existence of comorbid conditions (hypertension, diabetes mellitus, and hyperlipidemia) (Table S2–S4).

**Table 4:** The impact of total comprehensive stroke care (CSC) score on in-hospital mortality after ischemic stroke, adjusted by age, sex, and level of consciousness at admission according to the Japan Coma Scale (JCS).

Factor	OR	95% CI	P value
Male	1.23	1.12–1.35	<0.001
Age	1.40	1.34–1.47	<0.001
CSC total score	0.97	0.96–0.99	0.001
JCS			
normal	1		
one-digit code	2.40	2.11–2.74	<0.001
two-digit code	7.46	6.47–8.60	<0.001
three-digit code	21.62	18.69–25.02	<0.001

CI, confidence interval; CSC, comprehensive stroke care; JCS, Japan Coma Scale; OR, odds ratio.

**Table 5:** The impact of total comprehensive stroke care (CSC) score on in-hospital mortality after intracerebral hemorrhage, adjusted by age, sex, and level of consciousness at admission according to the Japan Coma Scale (JCS).

Factor	OR	95% CI	P value
Male	1.72	1.54–1.92	<0.001
Age	1.36	1.30–1.42	<0.001
CSC total score	0.97	0.95–0.99	0.003
JCS			
normal	1		
one-digit code	1.45	1.14–1.83	0.002
two-digit code	4.22	3.34–5.33	<0.001
three-digit code	49.59	40.12–61.27	<0.001

CI, confidence interval; CSC, comprehensive stroke care; JCS, Japan Coma Scale; OR, odds ratio.

**Table 6:** The impact of total comprehensive stroke care (CSC) score on in-hospital mortality after subarachnoid hemorrhage, adjusted by age, sex, and level of consciousness at admission according to the Japan Coma Scale (JCS).

Factor	OR	95%CI	P value
Male	1.39	1.17–1.65	<0.001
Age	1.37	1.29–1.45	<0.001
CSC total score	0.95	0.93–0.98	<0.001
JCS			
normal	1		
one-digit code	1.05	0.75–1.46	0.785
two-digit code	2.01	1.46–2.77	<0.001
three-digit code	17.13	13.14–22.35	<0.001

CI, confidence interval; CSC, comprehensive stroke care; JCS, Japan Coma Scale; OR, odds ratio.

Table 7–9 show the correlations between CSC subcategory scores and in-hospital mortality adjusted for age, sex, and level of consciousness depending on the different stroke types: mortality of patients with ischemic stroke was significantly correlated with subcategory scores in personnel (OR = 0.93), infrastructure (OR = 0.94), and education (OR = 0.89) (Table 7). Mortality of patients with ICH was significantly correlated with subcategory scores in diagnostic technique (OR = 0.91), infrastructure (OR = 0.92), and education (OR = 0.91) (Table 8). Mortality of patients with SAH was significantly associated with subcategory scores in personnel (OR = 0.91) specific expertise (OR = 0.83), infrastructure (OR = 0.89), and education (OR = 0.84) (Table 9). We found that while infrastructure and education subcategory CSC scores significantly impacted outcomes for all types of stroke, other subcategory CSC scores were differentially associated with in-hospital mortality depending on stroke type.

**Table 7:** The impact of subcategory CSC score on in-hospital mortality after ischemic stroke adjusted by age, sex and JCS.

Component	OR	95% CI	P value
Personnel	0.93	0.88–0.98	0.008
Diagnostic techniques	0.95	0.90–1.01	0.090
Specific expertise	0.96	0.90–1.01	0.136
Infrastructure	0.94	0.90–0.99	0.014
Education/research	0.89	0.83–0.96	0.003

CI, confidence interval; CSC, comprehensive stroke care; JCS, Japan Coma Scale; OR, odds ratio.

**Table 8:** The impact of subcategory CSC score on in-hospital mortality after intracerebral hemorrhage adjusted by age, sex and JCS.

Component	OR	95% CI	P value
Personnel	0.98	0.92–1.04	0.523
Diagnostic techniques	0.91	0.85–0.98	0.012
Specific expertise	0.93	0.86–1.00	0.055
Infrastructure	0.92	0.87–0.98	0.005
Education/research	0.91	0.83–1.00	0.047

CI, confidence interval; CSC, comprehensive stroke care; JCS, Japan Coma Scale; OR, odds ratio.

**Table 9:** The impact of subcategory CSC score on in-hospital mortality after subarachnoid hemorrhage adjusted by age, sex and JCS.

Component	OR	95% CI	P value
Personnel	0.91	0.84–0.98	0.016
Diagnostic techniques	1.01	0.92–1.11	0.896
Specific expertise	0.83	0.75–0.93	<0.001
Infrastructure	0.89	0.83–0.96	0.002
Education/research	0.84	0.75–0.95	0.005

CI, confidence interval; CSC, comprehensive stroke care; JCS, Japan Coma Scale; OR, odds ratio.

**Figure 1:**
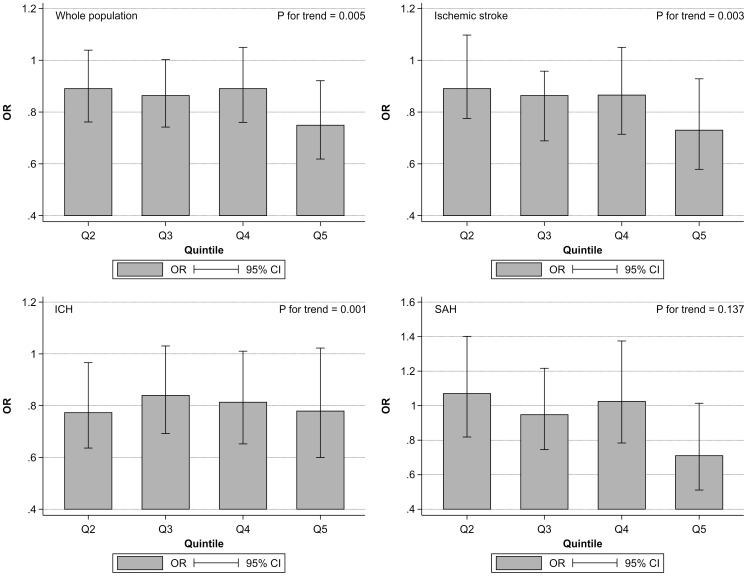
Associations between total comprehensive stroke care (CSC) scores separated into quintiles (Q1: 4–12, Q2: 13–14, Q3: 15–17, Q4: 18, Q5: 19–23) and in-hospital mortality of patients after all types of stroke (a), ischemic stroke (b), intracerebral hemorrhage (ICH) (c), and subarachnoid hemorrhage (SAH) (d), after adjustment for age and sex. Odds ratios (ORs) and 95% confidence intervals (CIs) of in-hospital mortality of each total CSC score quintile are depicted compared with that of Q1 as control.

**Figure 2:**
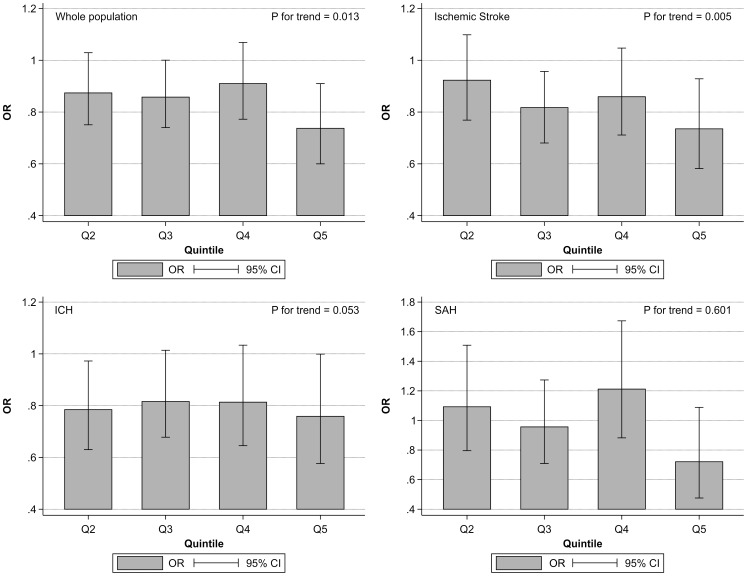
Associations between total comprehensive stroke care (CSC) scores separated into quintiles (Q1: 4–12, Q2: 13–14, Q3: 15–17, Q4: 18, Q5: 19–23) and in-hospital mortality of patients after all types of stroke (a), ischemic stroke (b), intracerebral hemorrhage (ICH) (c), and subarachnoid hemorrhage (SAH) (d), after adjustment for age; sex; initial level of consciousness; and incidence of hypertension, hyperlipidemia, and diabetes mellitus. Odds ratios (ORs) and 95% confidence intervals (CIs) of the in-hospital mortality of each total CSC score quintile are depicted compared with that of Q1 as control.

Figure 1 shows the impact of total CSC score classified into quintiles (Q1: 4–12, Q2: 13–14, Q3: 15–17, Q4: 18, Q5: 19–23) on the in-hospital mortality of patients with all types of stroke (a), ischemic stroke (b), ICH (c), and SAH (d) after adjusting for age, sex and level of consciousness. There was a significant association between total CSC score and in-hospital mortality in all types of stroke (all P<0.001) (Table 4–6). Figure 2 illustrates the impact of total CSC score on the in-hospital mortality of patients with all types of stroke (a), ischemic stroke (b), ICH (c), and SAH (d) after adjustment for age; sex; level of consciousness; and incidence of hypertension, hyperlipidemia, and diabetes mellitus. The association between total CSC score and in-hospital mortality in patients after all types of stroke (a), ischemic stroke (b), and ICH (c) remained significant after adjustment for age; sex; level of consciousness; and incidence of hypertension, hyperlipidemia, and diabetes mellitus. This same association was not evident in patients with SAH (P = 0.601) (Table 10).

**Table 10:** Associations between total comprehensive stroke care (CSC) scores separated into quintiles (Q1: 4–12, Q2: 13–14, Q3: 15–17, Q4: 18, Q5: 19–23) and in-hospital mortality of patients after all types of stroke (a), ischemic stroke (b), intracerebral hemorrhage (ICH) (c), and subarachnoid hemorrhage (SAH) (d), model 1: after adjustment for age, sex, and initial level of consciousness; and model 2: after adjustment for age, sex, initial level of consciousness, and incidence of hypertension, hyperlipidemia, and diabetes mellitus.

		Model 1					Model 2				
Type of Stroke	Quintile	OR	P value	95% CI		P for trend	OR	P value	95% CI		P for trend
Whole Population(n = 53,170)	Q2	0.87	0.077	0.74	1.02	0.005	0.88	0.119	0.75	1.03	0.013
	Q3	0.84	0.023	0.72	0.98		0.86	0.045	0.74	1.00	
	Q4	0.87	0.115	0.74	1.03		0.91	0.254	0.77	1.07	
	Q5	0.73	0.003	0.60	0.90		0.74	0.004	0.60	0.91	
Ischemic Stroke (n = 32,671)	Q2	0.90	0.278	0.75	1.08	0.003	0.92	0.356	0.77	1.10	0.005
	Q3	0.79	0.008	0.66	0.94		0.81	0.015	0.68	0.96	
	Q4	0.84	0.097	0.69	1.03		0.86	0.131	0.71	1.05	
	Q5	0.71	0.006	0.56	0.91		0.73	0.01	0.58	0.93	
ICH (n = 15,699)	Q2	0.76	0.015	0.62	0.95	<0.001	0.79	0.034	0.63	0.98	0.053
	Q3	0.82	0.058	0.67	1.01		0.83	0.083	0.68	1.02	
	Q4	0.79	0.039	0.63	0.99		0.82	0.099	0.65	1.04	
	Q5	0.76	0.050	0.58	1.00		0.76	0.051	0.58	1.00	
SAH (n = 4,934)	Q2	1.04	0.814	0.78	1.38	0.137	1.10	0.568	0.80	1.51	0.601
	Q3	0.92	0.524	0.71	1.19		0.96	0.767	0.71	1.28	
	Q4	1.00	0.975	0.75	1.34		1.22	0.232	0.88	1.68	
	Q5	0.68	0.043	0.47	0.99		0.73	0.126	0.48	1.09	

ICH, intracerebral hemorrhage; SAH, subarachnoid hemorrhage.

Hospitals with higher CSC scores were also more likely to provide early rehabilitation, improved monitoring, the possibility of admission to an SCU, presence of an acute stroke care team, existence of a t-PA protocol, greater numbers of t-PA cases/year, and higher scores on the organized stroke care index. In addition to the CSC score, the processes of acute stroke care, such as admission to SCU, presence of an acute stroke team, the organized stroke care index [7], and number of acute stroke cases/staff physician significantly impacted in-hospital mortality after all types of acute stroke, although in some cases, to a greater or lesser degree for the different types of stroke (Table 11–14).

**Table 11:** Impact of processes of stroke care on in-hospital mortality after all types of stroke.

	OR	P value	95% CI
Monitoring (%)	1.04	0.53	0.92–1.17
Early rehabilitation (%)	1.15	0.352	0.86–1.52
Admission to SCU (%)	0.87	0.039	0.76–0.99
Acute stroke team	0.88	0.029	0.79–0.99
Organized care index*	0.93	0.031	0.86–0.99
Existence of t-PA protocol (%)	0.88	0.295	0.69–1.12
Number of t-PA cases/year (mean)	1.00	0.203	0.99–1.00
Number of acute stroke patients/staff physician (mean)	0.999	0.012	0.998–1.000

*The organized stroke care index was created to represent different levels of access to organized stroke care ranging from 0 to 3 as determined by the presence of early rehabilitation, acute stroke team assessment, and admission to a stroke unit based on the report of Saposnik et al. (2009).

SCU, stroke care unit; t-PA, tissue plasminogen activator.

**Table 12:** Impact of processes of stroke care on in-hospital mortality after ischemic stroke.

	OR	P value	95% CI
Monitoring (%)	0.98	0.738	0.85–1.12
Early rehabilitation (%)	1.09	0.615	0.78–1.52
Admission to SCU (%)	0.91	0.218	0.78–1.06
Acute stroke team	0.85	0.016	0.74–0.97
Organized care index*	0.92	0.055	0.85–1.00
Existence of t-PA protocol (%)	0.82	0.158	0.61–1.08
Number of t-PA cases/year (mean)	0.99	0.132	0.98–1.00
Number of acute stroke patients/staff physician (mean)	0.999	0.047	0.998–1.000

*The organized stroke care index was created to represent different levels of access to organized stroke care ranging from 0 to 3 as determined by the presence of early rehabilitation, acute stroke team assessment, and admission to a stroke unit based on the report of Saposnik et al. (2009).

SCU, stroke care unit; t-PA, tissue plasminogen activator.

**Table 13:** Impact of processes of stroke care on in-hospital mortality after intracerebral hemorrhage.

	OR	P value	95% CI
Monitoring (%)	1.12	0.177	0.95–1.32
Early rehabilitation (%)	1.39	0.091	0.95–2.03
Hospitalization for SCU (%)	0.84	0.048	0.70–1.00
Acute stroke team	0.90	0.194	0.77–1.05
Organized care index*	0.93	0.163	0.85–1.03
Existence of t-PA protocol (%)	0.84	0.314	0.60–1.18
Number of t-PA cases/year (mean)	1.00	0.313	0.99–1.00
Number of acute stroke patients/staff physician (mean)	0.999	0.043	0.998–1.000

*The organized stroke care index was created to represent different levels of access to organized stroke care ranging from 0 to 3 as determined by the presence of early rehabilitation, acute stroke team assessment, and admission to a stroke unit based on the report of Saposnik et al. (2009).

SCU, stroke care unit; t-PA, tissue plasminogen activator.

**Table 14:** Impact of processes of stroke care on in-hospital mortality after subarachnoid hemorrhage.

	OR	P value	95% CI
Monitoring (%)	1.04	0.737	0.84–1.28
Early rehabilitation (%)	1.02	0.945	0.63–1.64
Admission to SCU (%)	0.79	0.039	0.63–0.99
Acute stroke team	0.85	0.101	0.69–1.03
Organized care index*	0.88	0.034	0.78–0.99
Existence of t-PA protocol (%)	1.09	0.732	0.66–1.81
Number of t-PA cases/year (mean)	1.00	0.456	0.98–1.01
Number of acute stroke patients/staff physician (mean)	0.998	0.006	0.997–1.000

*The organized stroke care index was created to represent different levels of access to organized stroke care ranging from 0 to 3 as determined by the presence of early rehabilitation, acute stroke team assessment, and admission to a stroke unit based on the report of Saposnik et al. (2009).

SCU, stroke care unit; t-PA, tissue plasminogen activator.

## Discussion

Using the nationwide discharge data obtained from the Japanese DPC-based Payment System, we evaluated the effect of hospital characteristics based on the recommended components of CSCs [2] on the in-hospital mortality of patients with acute ischemic and hemorrhagic stroke treated between April 1, 2010 and March 31, 2011. We found that the total CSC score was significantly associated with in-hospital mortality rates irrespective of stroke type after adjustment for age, sex, and initial level of consciousness according to the Japan Coma Scale. However, the subcategory scores that were significantly associated with in-hospital mortality differed among stroke type. Importantly, the association between total CSC scores and in-hospital mortality remained significant after adjustment for age; sex; initial level of consciousness according to the Japan Coma Scale; and incidence of hypertension, diabetes mellitus and hyperlipidemia for all types of stroke except SAH. These findings highlight the importance of CSC capabilities for optimal treatment of ischemic and hemorrhagic stroke and will enable health care professionals and policy makers to focus their efforts on improving specific aspects of CSC capabilities for different types of stroke.

Increasing attention has been given to defining the quality and value of health care through the reporting of process and outcome measures. Following the original proposal to establish CSCs [2], detailed metrics for measuring quality of care in CSCs have been reported [5]. The so-called “drip-and-ship” model has emerged as a paradigm for emergency departments that are able to diagnose acute ischemic stroke and administer intravenous (IV) recombinant t-PA (rt-PA) but lack the infrastructure to provide intensive monitoring for patients after rt-PA administration [15], [16]. A recent study demonstrated that despite having more severe strokes on arrival at the CSC, transfer-in patients with acute ischemic stroke had in-hospital mortality similar to that of front door patients and were more likely to be discharged to rehabilitation. These findings lend support to the concept of regionalized stroke care and directing patients with greater disability to more advanced stroke centers [17]. At present, no official certification of stroke centers in Japan has been launched, and the current study indicates that patients with acute ischemic stroke or hemorrhagic stroke are being admitted on an emergent basis to hospitals with similar CSC total and subcategory scores.

In the present study, stroke severity was adjusted by baseline level of consciousness according to the Japan Coma Scale [10], [11]. The Get With the Guidelines-Stroke (GWTG-Stroke) risk model was recently developed to predict in-hospital ischemic stroke mortality, suggesting that the National Institutes of Health Stroke Scale (NIHSS) score provides substantial incremental information on a patient's mortality risk [18], emphasizing the importance of adjustment of stroke severity to develop a hospital risk model for mortality [19]. Previous prospective multicenter study has demonstrated that the development of a decreased level of consciousness within the initial hours after stroke onset, as evaluated by a simple six-point scale, is a powerful independent predictor of mortality after a major ischemic stroke of the anterior vasculature [20]. In hemorrhagic stroke, the degree of impaired consciousness at admission was also included in the various proposed ICH scores to predict functional outcome and mortality [21], [22]. This study demonstrated that the level of consciousness at admission, as measured by the Japan Coma Scale, is a powerful independent predictor of mortality after ischemic and hemorrhagic stroke. Determining an individual patient's risk of mortality at admission could improve clinical care by providing valuable information to patients and their family members and by identifying those at high risk for poor outcomes who may require more intensive resources.

Health care quality of CSCs in the present study was scored on the basis of the results of a questionnaire referring to 25 items originally recommended by the Brain Attack Coalition. Although there is now increasingly good evidence from initiatives like GWTG-Stroke [23] that a process based on the systematic collection and evaluation of stroke performance measures can rapidly improve the quality of stroke care delivered by hospitals, current metrics are mostly limited to process measures that address the care of patients with ischemic stroke in acute hospital-based settings [4]. In addition, there is a pressing need to demonstrate a direct link between better adherence to stroke performance measures and improved patient-oriented outcomes [4], [24].

One potential issue with the interpretation of this study could be the lack of a control group. Although there is currently no clear consensus regarding the recommended criteria for CSCs in Japan, the present study distinctly shows that the CSC scores widely distributed in Figure 1 and classified into quintiles were significantly associated with in-hospital mortality for all types of stroke; in fact, mortality after all types of stroke markedly decreased, for example, in ischemic stroke cases by about 40% in hospitals in the highest quintile compared with those in the lowest quintile.

In the present study, our questionnaire was primarily based on the American definition of CSCs. However, according to the definitions derived from a European survey of experts in the field [25], facilities that meet the criteria for CSCs should include the capability to conduct sophisticated monitoring, such as automated electrocardiography (ECG) monitoring at bedside and automated monitoring of pulse oximetry, in addition to the numerous aspects of care capability indexed by the American definition of CSCs used in this study. According to the European approach, to meet the criteria for CSCs, hospitals should have the availability of at least 80% of the components rated as absolutely necessary by at least 50% of experts who participated in the previous expert survey; moreover, these components must be present in each of 6 categories and include the 19 components rated as absolutely necessary by >75% of experts. Based on the present results and an additional ongoing study using a validation cohort in Japan, the criteria for the designation of CSCs in Japan should be determined after further thorough discussion among Japanese stroke experts.

The present study demonstrated the feasibility and impact of using nationwide discharge data with hierarchical logistic regression analysis to examine the random effects that vary among hospitals, as well as the fixed effects of CSC score and patient effects of age, sex, and level of consciousness. We used unique hospital ID in random-intercept hierarchical regression models to assess the association between CSC score and mortality, adjusting for patient characteristics and the hospital where a patient was treated.

This model adjusts for hospital-level effects that arise from factors such as geographical location and ageing of the local population. After adjustment, we can isolate the pure CSC score effects on mortality by hospital, as discussed by Localio et al. [26]. If the CSC score is no longer significant after accounting for hospital-level variation, the differences in mortality can be assumed to arise from differences among hospitals. This approach enabled us to elucidate the impact of various CSC metrics on in-hospital mortality of patients with different stroke types. By expanding the scope of performance measures to include all types of stroke, the present study was able to direct links between specific recommended items of CSC capacities and in-hospital mortality after both hemorrhagic and ischemic stroke. While previous reports [7] showed that aspects of acute stroke care, such as admission to an SCU, the presence of an acute stroke team, and the organized stroke care index, were significantly associated with effects on in-hospital mortality after acute stroke, the present study clearly shows that the same is true for the CSC score based on the items recommended by the American Stroke Association.

Finally, one could argue that there really is no concept of 3/4 CSCs, but rather only CSCs or PSCs. In light of the existing evidence regarding the impact of the recommended CSC items on stroke outcomes, we advocate a CSC scoring system to examine the impact of availability of the recommended items on in-hospital mortality for all types of stroke. Considering the marked impact of the CSC score on outcome after all types of stroke, the differential impacts of CSC subcategory scores for different stroke types may make a single simple and effective CSC criterion unrealistic as a tool to produce a nationwide reduction in stroke mortality. In our opinion, it may be a more viable option to employ CSC scores in a more limited fashion, that is, to benchmark the state of care currently provided by medical centers treating stroke patients.

### Limitations

First, the questionnaire used in this study is new and has not undergone pilot testing or systematic analysis; thus, its validity and reliability are uncertain. The current set of CSC score items does not include all worthwhile items related to CSC. These items were appropriately modified from the original American version [2] in consideration of the medical, social, and logistical differences between the U.S. and Japan. For example, most cerebrovascular surgeries, including carotid endarterectomy and neurointervention, are performed by neurosurgeons rather than vascular surgeons or neuroradiologists. At the beginning of this project, a survey including the 25 components recommended by the Brain Attack Coalition in 2005 was created after a review of the literature on comprehensive stroke centers and a thorough discussion by an expert panel. Some recommended items such as availability of ventriculostomy were excluded from our questionnaire merely for simplicity, and thus to increase the response rate of the survey, since they seemed to be identical to recommendations of the board-certified neurosurgeons of Japan. On the other hand, some items such as transesophageal echocardiography were excluded because of the expected very low usage of this examination. According to the Japanese Stroke Databank 2009, for example, transesophageal echocardiography was performed for only 5.4% of the 34,417 acute ischemic stroke patients in a real-world situation. The impact of this examination on the mortality rates of this study would be difficult to evaluate because of low usage. All 25 components of the questionnaire are shown in the appendix. Second, discharge data obtained from the Japanese DPC-based Payment System lacks important information, such as post-discharge data and an NIHSS or GCS score as an index of stroke severity at admission. Although the Japan Coma Scale proved to be a powerful independent predictor of mortality after all types of stroke, further study is necessary to validate the results of the present study with another indicator of stroke severity, such as the NIHSS or GCS. This can be done separately or in combination with a validation data set (the estimated data volume in the J-ASPECT 2013 is more than 80,000 cases that will be available in 2013). Third, there is an inherent risk of information bias when evaluating data obtained by self-assessment. Moreover, hospitals actively working to improve stroke care may have been more likely to respond to the questionnaire. Admittedly, the participation rate of DPC data collection of the J-ASPECT study was relatively low. The participation rates were 19.4% of the 1,369 certified training institutions of the Japan Neurosurgical Society, and 35.4% of the 749 hospitals that responded to the institutional survey. However, the institutes that participated in the present study tended to have more beds and more stroke cases than average. This suggests that the hospitals that were more active in stroke care and potentially eligible for CSC participated in this study. Therefore, the present results may not generalize to non-participating hospitals. External validation greatly increases the reliability of self-assessment data. Accordingly, we plan to validate the information regarding hospital characteristics and outcomes by using a small sample set from the 2010 validation cohort of the present study. Since the number of participating hospitals in this study is increasing every year, we are planning to evaluate how the validity and reliability of the CSC score in predicting stroke patient mortality changes when weighting factors are applied to the recommended items, stroke type, and severity. Through annual evaluations, we aim to achieve higher predictive validity and responsiveness to establish the usefulness of the CSC score. Fourth, we assigned 1 point if the hospital met each recommended item for CSC. However, this equal weight assumption is probably not valid since some components were not significant on subgroup analysis. Although some associations between individual CSC components and mortality achieved significance, several did not but were very close to significant, based on the confidence intervals. These non-significant trends are telling and suggest that the subgroup component analyses were underpowered and thus prone to type 2 error. In addition, we performed multiple comparisons for each stroke type, and therefore, some of the secondary analyses, particularly those that evaluated the impact of stroke care procedures on in-hospital mortality, are prone to type 1 error. We need a larger sample size to validate each recommended item and appropriately weight the subscores.

## Conclusions

Although patient demographics and stroke severity are important predictors of in-hospital mortality of patients with all types of stroke, CSC capacities were associated with reduced in-hospital mortality rates, with relevant aspects of care dependent on stroke type.

## Supporting Information

File S1 English translation of the survey.
(DOCX)

File S2 List of the participating hospitals (J-ASPECT Study).
(DOCX)

Table S1 Japan Coma Scale for grading impaired consciousness.
(DOCX)

Table S2 The impact of total comprehensive stroke care (CSC) score on in-hospital mortality after ischemic stroke adjusted by age, sex, level of consciousness at admission, and incidence of hypertension (HTN), diabetes mellitus(DM), and hyperlipidemia(HPL).
(DOCX)

Table S3 The impact of total comprehensive stroke care (CSC) score on in-hospital mortality after intracerebral hemorrhage adjusted by age, sex, level of consciousness at admission, and incidence of hypertension (HTN), diabetes mellitus(DM), and hyperlipidemia(HPL).
(DOCX)

Table S4 The impact of total comprehensive stroke care (CSC) score on in-hospital mortality after subarachnoid hemorrhage adjusted by age, sex, level of consciousness at admission, and incidence of hypertension (HTN), diabetes mellitus(DM), and hyperlipidemia(HPL).
(DOCX)
