# Supplementary material for: Effects of Comprehensive Stroke Care Capabilities on In-Hospital Mortality of Patients with Ischemic and Hemorrhagic Stroke: J-ASPECT Study
Source: PLoS One. 2014 May 14;9(5):e96819. doi: 10.1371/journal.pone.0096819 (PMC4020787; doi:10.1371/journal.pone.0096819)
Supplement: File S1 — English translation of the survey. (DOCX) [file pone.0096819.s001.docx]

File S1. The English version of the survey.

# A : About stroke professionals

#### How many certified physicians does your facility have according to the age? （If the same persons more than one qualification, please count each qualification as one person.）

|  | ≤39 | 40〜49 | 50〜59 | 60〜69 | 70≤ |
| --- | --- | --- | --- | --- | --- |
| The Japan Neurosurgical Society certified surgeons |  |  |  |  |  |
| Societas Neurologica Japonica certified physician |  |  |  |  |  |
| Japanese Association for Acute Medicine certified physicians |  |  |  |  |  |
| The Japanese Association of Rehabilitation Medicine certified physicians |  |  |  |  |  |
| The Japan Stroke Society Certified physicians |  |  |  |  |  |
| The Japanese Society for Neuroendovascular Therapy Certified physicians |  |  |  |  |  |

#### How many doctors can perform therapies as a chief operator in your facility? Please fill in the numbers according to each therapy.

| 1. | Intravenous t-PA |  |
| --- | --- | --- |
| 2. | Intravenous thrombolytic therapy/percutaneous angioplasty |  |
| 3. | Carotid endarterectomy |  |
| 4. | Carotid Artery Stenting |  |
| 5. | Extracranial-intracranial bypass |  |
| 6. | Clipping of the cerebral aneurysm |  |
| 7. | Coiling of the cerebral aneurysm |  |
| 8. | Craniotomy for evacuation of intracerebral hematomas |  |
| 9. | **Stereotactic evacuation** Stereotactic evacuation of intracerebral hematomas |  |
| 10. | Endoscopic evacuation of intracerebral hematomas |  |

#### Dose your facility provide an acute stroke team, which is consisted by different types of specialists?

GO TO Q5

1. No
2. Yes

GO TO Q4

**【If your facility provide acute stroke team】**

#### How many specialists does the acute stroke team consist of? （If the same persons more than one qualification, please count each qualification as one person.）

| 1. | The Japan Neurosurgical Society certified surgeons | persons |
| --- | --- | --- |
| 2. | The Japan Stroke Society certified physicians | persons |
| 3. | The Japanese Society for Neuroendovascular Therapy certified physicians | persons |
| 4. | Societas Neurologica Japonica certified physicians | persons |
| 5. | Japanese Association for Acute Medicine certified physicians | persons |
| 6. | Residents attending stroke care who are members of any society of above 1-5 | persons |
| 7. | Radiologist/Neuroradiologist | persons |
| 8. | The Japanese Association of Rehabilitation Medicine certified physicians | persons |
| 9. | Physical Therapist（PT） | persons |
| 10. | Occupational Therapist（OT） | persons |
| 11. | Speech Therapist（ST） | persons |

#### Dose your facility provide Stroke Care Unit / Stroke Unit （SCU / SU）？

GO TO Q6

1. No
2. Yes

GO TO Q7

**【If your facility dose not provide SCU / SU】**

#### In which ward do your facility care acute stroke patients?

| 1. | Intensive care Unit（ICU） |
| --- | --- |
| 2. | High Care Unit（HCU） |
| 3. | General ward |

#### Does your facility implement facility criterion about stroke care unit inpatient management fee?

| 0. | No | 1. | Yes |
| --- | --- | --- | --- |

# C : About rehabilitation

#### Dose your facility have nurses on stroke rehabilitation?

| 0. | No | 1. | Yes |
| --- | --- | --- | --- |

#### Dose your facility have physical therapists (PT)?

| 0. | NO | 1. | Yes |
| --- | --- | --- | --- |

#### Dose your facility have occupational therapists (OT)?

| 0. | No | 1. | Yes |
| --- | --- | --- | --- |

#### Dose your facility have speech therapists (ST)?

| 0. | No | 1. | Yes |
| --- | --- | --- | --- |

# D : About implementation system for imaging test

#### The following question to ask about implementation system for imaging test. Circle the most appropriate item which fit into your facility.

|  |  | Not available | Available within office hours | Available at all hours |
| --- | --- | --- | --- | --- |
| A） | CT | 1 | 2 | 3 |
| B） | MRI (including Diffusion Weighted Image) | 1 | 2 | 3 |
| C） | Digital Subtraction Angiography | 1 | 2 | 3 |
| D） | CT Angiography | 1 | 2 | 3 |
| E） | Carotid artery ultrasound | 1 | 2 | 3 |
| F） | Transcranial doppler | 1 | 2 | 3 |

#### Does your facility have a stroke registry?

0. NO

1. Yes

# E : About educational programs

#### Does your facility hold educational workshop for health-care providers engaged in a stroke care?

| 0. | No | 1. | Yes |
| --- | --- | --- | --- |

#### Does your facility hold educational workshop for emergency services?

| 0. | No | 1. | Yes |
| --- | --- | --- | --- |

#### Does your facility hold educational workshop for general population?

| 0. | No | 1. | Yes |
| --- | --- | --- | --- |

#### If advanced surgery for acute stroke will be needed, what do you do?

| 1. | Inviting a specialist from other hospitals | 3. | Operating with your staff |
| --- | --- | --- | --- |
| 2. | Taking a patient to the nearest stroke center | 4. | Others |

#### If endovascular surgery for acute stroke will be needed, what do you do?

| 1. | Inviting a specialist from other hospitals | 3. | Operating with your staff |
| --- | --- | --- | --- |
| 2. | Taking a patient to the nearest stroke center | 4. | Others |
