# Supplementary material for: Effects of Comprehensive Stroke Care Capabilities on In-Hospital Mortality of Patients with Ischemic and Hemorrhagic Stroke: J-ASPECT Study
Source: PLoS One. 2014 May 14;9(5):e96819. doi: 10.1371/journal.pone.0096819 (PMC4020787; doi:10.1371/journal.pone.0096819)
Supplement: File S2 — List of the participating hospitals (J-ASPECT Study). (DOCX) [file pone.0096819.s002.docx]

File S2. List of the participating hospitals (J-ASPECT Study)

Abashiri Neurosurgical Rehabilitation Hospital, Akita General Hospital, Almeida Memorial Hospital, Arao City Hospital, Asahikawa Medical University Hospital, Asahikawa Red Cross Hospital, Ayabe City Hospital, Azumi General Hospital, Azumino Red Cross Hospital, Bellland General Hospital, Chikamori Hospital, Daini Okamoto General Hospital , Dohtoh Neurosurgical Hospital, Ehime Prefectural Central Hospital, Ehime University Hospital, Fuchu Hospital, Fuji Brain Institute and Hospital, Fuji City General Hospital, Fuji Neurosurgical Hospital, Fujita Health University Hospital, Fujiyoshida Municipal Medical Center, Fukui University Hospital, Fukuoka City Hospital, Fukuoka Seishukai Hospital, Fukuoka Tokushukai Medical Center, Fukuroi Municipal Hospital, Fukushima Red Cross Hospital, Gifu Municipal Hospital, Gifu University Hospital, Hakodate Neurosurgical Hospital, Hakodate Shintoshi Hospital, Hanwa Memorial Hospital, Hata Kenmin Hospital, Hayashi Hospital, Health Insurance Nankai Hospital, Higashiyamato Hospital, Hirosaki University Hospital, Hiroshima City Asa Hospital, Hiroshima Prefectural Hospital, Hiroshima Red Cross Hospital & Atomic-bomb Survivors Hospital, Hiroshima University Hospital, Hokkaido University Hospital, Hokuto Hospital, Hoshigaoka Koseinenkin Hospital, Houetsu Hospital, Hyogo Brain and Heart Center, Hyogo Prefectural Amagasaki Hospital, Hyogo Prefectural Nishinomiya Hospital, Ibaraki Seinan Medical Center Hospital, Iida Municipal Hospital, Inagi Municipal Hospital, Isesaki Municipal Hospital, Ishikawa Prefectural Central Hospital, Ishinkai Yao General Hospital, Itsukaichi Memorial Hospital, Iwate Medical University Hospital, Iwate Prefectural Iwai Hospital, Iwate Prefectural Ninohe Hospital, Izumino Hospital, Japanese Red Cross Kyoto Daini Hospital, Japanese Red Cross Medical Center, Japanese Red Cross Society Hachinohe Medical Center, Japanese Red Cross Society Himeji Hospital, Juntendo University Nerima Hospital, Juntendo University Urayasu Hospital, Junwakai Kinen Hospital, Juzenkai Hospital, Jyuzen General Hospital, Kaga City Hospital, Kagawa Prefectural Central Hospital, Kagawa Rosai Hospital, Kagoshima City Hospital, Kainan Hospital, Kakogawa City Hospital, Kansai Medical University Takii Hospital, Kasaoka Daiichi Hospital, Kashiwaba Neurosurgical Hospital, Kawachi General Hospital, Kawakita General Hospital, Kimitsu Chuo Hospital, Kobari General Hospital, Kobe University Hospital, Kochi Medical School Hospital, Kohka Public Hospital, Kokura Memorial Hospital, Komaki City Hospital, Komatsu Municipal Hospital, Koto Memorial Hospital, Kousei General Hospital, Kumamoto Red Cross Hospital, Kumamoto Rousai Hospital, Kumamoto University Hospital, Kurashiki Central Hospital, Kurashiki Heisei Hospital, Kure Kyosai Hospital, Kurosawa Hospital, Kurume University Hospital, Kuwana Hospital, Kyorin University Hospital, Kyoritsu General Hospital, Kyoto City Hospital, Kyoto Min-iren Chuo Hospital, Kyushu University Hospital, Maebashi Red Cross Hospital, Matsudo City Hospital, Matsushita Memorial Hospital, Matsuyama Shimin Hospital, Mazda Hospital, Meitetsu Hospital, Midorigaoka Hospital, Mihara Memorial Hospital, Minamata City General Hospital and Medical Center, Mito Medical Center, Mito Saiseikai General Hospital, Mitoyo General Hospital, Mitsugi General Hospital, Mizushima Central Hospital, Morioka Red Cross Hospital, Muroran City General Hospital, Nagahama City Hospital, Nagano Municipal Hospital, Nagano Prefectural Kiso Hospital, Nagaoka Chuo General Hospital, Nagasaki Kawatana Medical Center, Nagasaki Municipal Hospital, Nagasaki Prefectural Shimabara Hospital, Nagasaki University Hospital, Nagoya Medical Center, Nakamura Memorial Hospital, Nakano General Hospital, Nara Medical University Hospital, Nara Prefectural Hospital, Narita Red Cross Hospital, National Cerebral and Cardiovascular Center, National Fukuoka-Higashi Medical Center, National Hospital Organization Chiba Medical Center, National Hospital Organization Himeji Medical Center, National Hospital Organization Kanazawa Medical Center, National Hospital Organization Kanmon Medical Center, National Hospital Organization Kobe Medical Center, National Hospital Organization Kure Medical Center, National Hospital Organization Minami Wakayama Medical Center, National Hospital Organization Osaka Minami Medical Center, National Hospital Organization Shizuoka Medical Center, National Hospital Organization Ureshino Medical Center, Nayoro City General Hospital, Niigata Minami Hospital, Niigata Prefectural Central Hospital, Niigata Prefectural Tokamachi Hospital, Niigata University Medical & Dental Hospital, Nippon Medical School Chiba Hokusoh Hospital, Nishi-Agatsuma Welfare Hospital, Nishinomiya Kyoritsu Neurosurgical Hospital, Nishiwaki Municipal Hospital, Obara Hospital, Obihiro-Kosei General Hospital, Odate Municipal General Hospital, Ohnishi Neurological Center, Ohta General Hospital, Oita-Oka-Hospital, Okayama Kyokuto Hospital, Okayama University Hospital, Okazaki City Hospital, Okinawa Prefectural Miyako Hospital, Okinawa Prefectural Nanbu Medical Center and Children's Medical Center, Okinawa Prefectural Yaeyama Hospital, Okitama Public General Hospital, Omihachiman Community Medical Center, Omori Red Cross Hospital, Omuta City Hospital, Osaka Kosei-Nenkin Hospital, Osaka Neurological Institute, Osaka Police Hospital, Osaka Red Cross Hospital, Osaka University Hospital, Ota Memorial Hospital, Saga Prefectural Hospital Koseikan, Sagamihara Kyodo Hospital, Saiseikai Central Hospital, Saiseikai Fukuoka General Hospital, Saiseikai Gose Hospital, Saiseikai Nagasaki Hospital, Saiseikai Nakatsu Hospital, Saiseikai Noe Hospital, Saiseikai Toyama Hospital, Saiseikai Yamaguchi Hospital, Saiseikai Yokohanashi Tobu Hospital, Saitama Cardiovascular and Respiratory Center, Sakai City Hospital, Sankoukai Miyazaki Hospital, Sapporo City General Hospital, Sapporo Higashi-Tokushukai Hospital, Sasebo City General Hospital, Seguchi Neurosurgery Hospital, Seirei Memorial Hospital, Seirei Mikatagahara General Hospital, Sendai City Hospital, Shakaihoken Kobe Central Hospital, Shakaihoken Shimonoseki Kosei Hospital, Shiga University of Medical Science Hospital, Shimane University Hospital, Shin Koga Hospital, Shin-Tokyo Hospital, Shingu Municipal Medical Center, Shinko Hospital, Shinonoi General Hospital, Shinrakuen Hospital, Shinseikai Toyama Hospital, Shizuoka General Hospital, Shonan Kamakura General Hospital, Showa Inan General Hospital, Social Insurance Chuo General Hospital, Southern Tohoku General Hospital, Steel Memorial Hirohata Hospital, Suiseikai Kajikawa Hospital, Suwa Central Hospital, Suzuka Kaisei Hospital, Takamatsu Red Cross Hospital, Takarazuka City Hospital, Takatsuki General Hospital, Tane General Hospital, Tannan Regional Medical Center, Tanushimaru Central Hospital, Tenshindo Hetsugi Hospital, The Taiju-Kai Foundation Social Medical Corporation, Kaisei General Hospital, Tochigi National Hospital, Toki General Hospital, Tokuda Neurosurgical Hospital, Tokushima University Hospital, Tokyo Metropolitan Health and Medical Corporation Toshima Hospital, Tonami General Hospital, Tosei General Hospital, Tottori University Hospital, Toyokawa City Hospital, Tsuchiura Kyodo General Hospital, Tsukuba Medical Center Hospital, Tsushima City Hospital, Tsuyama Central Hospital, "Ube Industries, Ltd. Central Hospital", Ugadake Hospital, University Hospital of the Ryukyus, University of Yamanashi Hospital, Urasoe General Hospital, Ushioda General Hospital, Uwajima City Hospital, Veritas Hospital, Wada Hospital, Wakakusa Dai-ichi Hospital, Yahata General Hospital, Yamagata City Hospital Saiseikan, Yamagata Prefectural Shinjo Hospital, Yamaguchi Grand Medical Center, Yamanashi Prefectural Central Hospital, Yamashiro Public Hospital, Yasugi Municipal Hospital, Yatsushiro Health Insurance General Hospital, Yawata Medical Center, Yokkaichi Municipal Hospital, Yokohama City Minato Red Cross Hospital, Yokohama City University Hospital, Yokohama Sakae Kyosai Hospital, Yokohama Shin-midori General Hospital, Yokohamashintoshi Neurosurgical Hospital, Yonago Medical Center, Yuaikai Hospital
