# Supplementary material for: Effects of Comprehensive Stroke Care Capabilities on In-Hospital Mortality of Patients with Ischemic and Hemorrhagic Stroke: J-ASPECT Study
Source: PLoS One. 2014 May 14;9(5):e96819. doi: 10.1371/journal.pone.0096819 (PMC4020787; doi:10.1371/journal.pone.0096819)
Supplement: Table S1 — Japan Coma Scale for grading impaired consciousness. (DOCX) [file pone.0096819.s003.docx]

Table S1. Japan Coma Scale for grading impaired consciousness.

| Grade | Consciousness Level |
| --- | --- |
| 1-digit code | The patient is awake without any stimuli and is: |
| 1 | Almost fully conscious |
| 2 | Unable to recognize time, place, and person |
| 3 | Unable to recall name or date of birth |
| 2-digit code | The patient can be aroused (then reverts to previous state after cessation of stimulation): |
| 10 | Easily by being spoken to (or is responsive with purposeful movements, phrases, or words)† |
| 20 | With loud voice or shaking of shoulders (or is almost always responsive to very simple words like yes or no or to movements)† |
| 30 | Only by repeated mechanical stimuli |
| 3-digit code | The patient cannot be aroused with forceful mechanical stimulation and |
| 100 | Responds with movements to avoid the stimulus |
| 200 | Responds with slight movements, including decerebrate and decorticate posture |
| 300 | Does not respond at all except for respiratory rhythm changes |

"R" and "I" are added to the grade to indicate restlessness and incontinence of urine and feces, respectively (e.g., 100-R and 30-RI).

†Criteria in parentheses are used in patients who do not open their eyes for any reason.
