# Supplementary material for: Effects of Comprehensive Stroke Care Capabilities on In-Hospital Mortality of Patients with Ischemic and Hemorrhagic Stroke: J-ASPECT Study
Source: PLoS One. 2014 May 14;9(5):e96819. doi: 10.1371/journal.pone.0096819 (PMC4020787; doi:10.1371/journal.pone.0096819)
Supplement: Table S2 — The impact of total comprehensive stroke care (CSC) score on in-hospital mortality after ischemic stroke adjusted by age, sex, level of consciousness at admission, and incidence of hypertension (HTN), diabetes mellitus(DM), and hyperlipidemia(HPL). (DOCX) [file pone.0096819.s004.docx]

Table S2. The impact of total comprehensive stroke care (CSC) score on in-hospital mortality after ischemic stroke adjusted by age, sex, level of consciousness at admission, and incidence of hypertension (HTN), diabetes mellitus(DM), and hyperlipidemia(HPL).

| Factor | OR | 95% CI | P value |
| --- | --- | --- | --- |
| Male | 1.18 | 1.07–1.29 | <0.001 |
| Age | 1.36 | 1.30–1.42 | <0.001 |
| CSC total score | 0.98 | 0.96–0.99 | 0.004 |
| JCS |  |  |  |
| normal | 1 |  |  |
| one-digit code | 2.28 | 2.00–2.59 | <0.001 |
| two-digit code | 6.60 | 5.73–7.62 | <0.001 |
| three-digit code | 18.78 | 16.21–21.76 | <0.001 |
| HTN | 1.13 | 1.03–1.25 | 0.014 |
| DM | 1.29 | 1.17–1.43 | <0.001 |
| HL | 0.37 | 0.33–0.42 | <0.001 |

JCS, Japan Coma Scale
