# Supplementary material for: Effects of Comprehensive Stroke Care Capabilities on In-Hospital Mortality of Patients with Ischemic and Hemorrhagic Stroke: J-ASPECT Study
Source: PLoS One. 2014 May 14;9(5):e96819. doi: 10.1371/journal.pone.0096819 (PMC4020787; doi:10.1371/journal.pone.0096819)
Supplement: Table S3 — The impact of total comprehensive stroke care (CSC) score on in-hospital mortality after intracerebral hemorrhage adjusted by age, sex, level of consciousness at admission, and incidence of hypertension (HTN), diabetes mellitus(DM), and hyperlipidemia(HPL). (DOCX) [file pone.0096819.s005.docx]

Table S3. The impact of total comprehensive stroke care (CSC) score on in-hospital mortality after intracerebral hemorrhage adjusted by age, sex, level of consciousness at admission, and incidence of hypertension (HTN), diabetes mellitus(DM), and hyperlipidemia(HPL).

| Factor | OR | 95% CI | P value |
| --- | --- | --- | --- |
| Male | 1.68 | 1.50–1.89 | <0.001 |
| Age | 1.33 | 1.28–1.39 | <0.001 |
| CSC total score | 0.98 | 0.96–1.00 | 0.016 |
| JCS |  |  |  |
| normal | 1 |  |  |
| one-digit code | 1.57 | 1.24–1.99 | <0.001 |
| two-digit code | 4.91 | 3.87–6.23 | <0.001 |
| three-digit code | 52.8 | 42.54–65.55 | <0.001 |
| HTN | 0.45 | 0.39–0.52 | <0.001 |
| DM | 0.91 | 0.80–1.04 | 0.171 |
| HL | 0.37 | 0.30–0.46 | <0.001 |

JCS, Japan Coma Scale
