# Supplementary material for: Effects of Comprehensive Stroke Care Capabilities on In-Hospital Mortality of Patients with Ischemic and Hemorrhagic Stroke: J-ASPECT Study
Source: PLoS One. 2014 May 14;9(5):e96819. doi: 10.1371/journal.pone.0096819 (PMC4020787; doi:10.1371/journal.pone.0096819)
Supplement: Table S4 — The impact of total comprehensive stroke care (CSC) score on in-hospital mortality after subarachnoid hemorrhage adjusted by age, sex, level of consciousness at admission, and incidence of hypertension (HTN), diabetes mellitus(DM), and hyperlipidemia(HPL). (DOCX) [file pone.0096819.s006.docx]

Table S4. The impact of total comprehensive stroke care (CSC) score on in-hospital mortality after subarachnoid hemorrhage adjusted by age, sex, level of consciousness at admission, and incidence of hypertension (HTN), diabetes mellitus(DM), and hyperlipidemia(HPL).

| Factor | OR | 95% CI | P value |
| --- | --- | --- | --- |
| Male | 1.35 | 1.12–1.63 | 0.002 |
| Age | 1.40 | 1.32–1.49 | <0.001 |
| CSC total score | 0.98 | 0.95–1.01 | 0.217 |
| JCS |  |  |  |
| normal | 1 |  |  |
| one-digit code | 1.28 | 0.91–1.82 | 0.161 |
| two-digit code | 2.63 | 1.88–3.69 | <0.001 |
| three-digit code | 16.44 | 12.40–21.8 | <0.001 |
| HTN | 0.20 | 0.16–0.25 | <0.001 |
| DM | 0.69 | 0.56–0.85 | <0.001 |
| HL | 0.20 | 0.16–0.25 | <0.001 |

JCS, Japan Coma Scale
